# International Rickettsia Disease Surveillance: An Example of Cooperative Research to Increase Laboratory Capability and Capacity for Risk Assessment of Rickettsial Outbreaks Worldwide

**DOI:** 10.3389/fmed.2021.622015

**Published:** 2021-03-02

**Authors:** Ju Jiang, Christina M. Farris, Kenneth B. Yeh, Allen L. Richards

**Affiliations:** ^1^Viral and Rickettsial Diseases Department, Naval Medical Research Center, Silver Spring, MD, United States; ^2^The Henry M. Jackson Foundation for the Advancement of Military Medicine, Inc., Bethesda, MD, United States; ^3^MRIGlobal, Gaithersburg, MD, United States; ^4^Department of Preventive Medicine and Biostatistics, Uniformed Services University of the Health Sciences, Bethesda, MD, United States

**Keywords:** rickettsioses, scrub typhus, cooperative international research, surveillance, orientia

## Abstract

Cooperative research that addresses infectious disease surveillance and outbreak investigations relies heavily on availability and effective use of appropriate diagnostic tools, including serological and molecular assays, as exemplified by the current COVID-19 pandemic. In this paper, we stress the importance of using these assays to support collaborative epidemiological studies to assess risk of rickettsial disease outbreaks among international partner countries. Workforce development, mentorship, and training are important components in building laboratory capability and capacity to assess risk of and mitigate emerging disease outbreaks. International partnerships that fund cooperative research through mentoring and on-the-job training are successful examples for enhancing infectious disease surveillance. Cooperative research studies between the Naval Medical Research Center's Rickettsial Diseases Research Program (RDRP) and 17 institutes from nine countries among five continents were conducted to address the presence of and the risk for endemic rickettsial diseases. To establish serological and molecular assays in the collaborative institutes, initial training and continued material, and technical support were provided by RDRP. The laboratory methods used in the research studies to detect and identify the rickettsial infections included (1) group-specific IgM and IgG serological assays and (2) molecular assays. Twenty-six cooperative research projects performed between 2008 and 2020 enhanced the capability and capacity of 17 research institutes to estimate risk of rickettsial diseases. These international collaborative studies have led to the recognition and/or confirmation of rickettsial diseases within each of the partner countries. In addition, with the identification of specific pathogen and non-pathogen *Rickettsia* species, a more accurate risk assessment could be made in surveillance studies using environmental samples. The discoveries from these projects reinforced international cooperation benefiting not only the partner countries but also the scientific community at large through presentations (*n* = 40) at international scientific meetings and peer-reviewed publications (*n* = 18). The cooperative research studies conducted in multiple international institutes led to the incorporation of new SOPs and trainings for laboratory procedures; biosafety, biosurety, and biosecurity methods; performance of rickettsia-specific assays; and the identification of known and unknown rickettsial agents through the introduction of new serologic and molecular assays that complemented traditional microbiology methods.

## Introduction

Rickettsial diseases are vector-borne diseases caused by agents of the genus *Rickettsia* (1, 2). However, the definition of rickettsial diseases can also be more inclusive to include diseases caused by agents that are genetically related to *Rickettsia*, such as *Orientia* species of the family Rickettsiaceae, and *Anaplasma, Ehrlichia*, and *Neorickettsia* species of the family Anaplasmataceae (3). Both families, Rickettsiaceae and Anaplasmataceae, are members of the order Rickettsiales within the class Alphaproteobacteria and phylum Proteobacteria. Lastly, there are some diseases such as Q fever and trench fever that are often associated with rickettsial diseases because the causative agents at one time were considered *Rickettsia* species (i.e., *Coxiella burnetii–Rickettsia burnetii* and *Bartonella quintana–Rickettsia quintana*, respectively) (3, 4). For the purposes of this report, rickettsial diseases will be limited to those diseases caused by *Rickettsia* and *Orientia* species.

Rickettsial diseases (and their causative agents) have been traditionally separated into three major groups based on their disease presentation, antigenicity, and vectors (Table 1). Those groups include the **typhus group** (epidemic typhus [*Rickettsia prowazekii*] and murine typhus [*Rickettsia typhi*]); **spotted fever group** (Rocky Mountain spotted fever (RMSF) [*Rickettsia rickettsii*], Mediterranean spotted fever (MSF) [*Rickettsia conorii*], African tick-bite fever [*Rickettsia africae*], flea-borne spotted fever [*Rickettsia felis*], Queensland tick typhus [*Rickettsia australis*], Japanese spotted fever [*Rickettsia japonica*], etc.), and **scrub typhus group** (scrub typhus [*Orientia tsutsugamushi, Candidatus* Orientia chuto, and *Candidatus* Orientia chiloensis]) (4–7) (Table 1). Genotyping of pathogenic and non-pathogenic rickettsial agents have led to over a dozen genogroups (8). These genogroups are not addressed herein.

**Table 1:** Three major groups of rickettsial diseases exist based on their causative agents, host seroreactivity to group-specific antigens, arthropod vectors, and their distribution.

**Diseases**	**Etiologic agents**	Serologic reactivity to antigens from^***^	**Vectors**	**Distribution**
**Typhus Group (TG)**				
Epidemic Typhus	*Rickettsia prowazekii*	TGR	*Pediculus humanus corporis—*human body louse; *Glaucomys volans—*flying squirrel ectoparasites	Worldwide
Murine Typhus	*Rickettsial typhi*	TGR	*Xenopsylla cheopis* Oriental rat flea	Worldwide
Spotted Fever Group (SFG)^*^^,^ ^**^				
Rocky Mountain spotted fever (RMSF)	*Rickettsia rickettsii*	SFGR	*Dermacentor variabilis Dermacentor andersoni Rhipicephalus sanguineus Amblyomma cajennense Amblyomma aureolatum*	Western Hemisphere
Mediterranean spotted fever (MSF)	*Rickettsia conorii* subsp. *conorii*	SFGR	*Rhipicehpalus sanguineus* Brown dog tick	Europe, northern Africa, western and southern Asia
Scalp eschar and neck lymphadenopathy after tick bite (SENLAT)	*Rickettsia slovaca* *Rickettsia raoultii* *Candidatus* Rickettsia rioja	SFGR	*Dermacentor marginatus Dermacentor reticulatus*	Europe and Central Asia
African tick-bite fever (ATBF)	*Rickettsia africae*	SFGR	*Amblyomma variegatum Amblyomma hebraeum*	Sub-Saharan Africa and Caribbean Islands
Japanese spotted fever (JSF)	*Rickettsia japonica*	SFGR	*Haemaphysalis longicornis, Haemaphysalis flava, Dermancentor taiwanensis, Ixodes ovatus*	Japan and Asia
Queensland tick typhus (QTT)	*Rickettsia australis*	SFGR	*Ixodes holocyclus, Ixodes tasmania*	Australia
Flea-borne spotted fever (FBSF)	*Rickettsia felis*	SFGR	*Ctenocephalides felis* Cat flea	Worldwide
Rickettsialpox	*Rickettsia akari*	SFGR	*Liponyssoides sanguineus* House mouse mite	Eastern Europe and Northeastern USA
**Scrub Typhus Group (STG)**				
Scrub typhus	*Orientia tsutsugamushi*	STGO	*Leptrombidum* species Trombiculid mites	Asia, Australia, and Islands of Indian and Pacific Oceans
Scrub typhus	*Candidatus* Orientia chuto	STGO	*Microtrombicula natalensis* Trombiculid mites	United Arab Emirates, Africa
Scrub typhus	*Candidatus* Orientia chiloensis	STGO	*Herpetacarus* species Trombiculid mites	Chile

* *Other SFG diseases and causative agent(s) include Israeli spotted fever (R. conorii subsp. israelensis), Astrakhan spotted fever (R. conorii subsp. caspia), Indian tick typhus (R. conorii subsp. Indica), Tidewater spotted fever (R. parkeri), Siberian tick typhus (R. sibirica), Lymphangitis-associated rickettsiosis (R. sibirica mongolitimonae), Aneruptive fever (R. helvetica), Far eastern tick-borne rickettsiosis (R. heilongjiangensis), and Flinders Island spotted fever (R. honei)*.

** *Potential SFG rickettsial pathogens include R. monacensis, R. aeschlimannii, R. massiliae, and R. asembonensis*.

*** *TGR, typhus group rickettsiae; SFGR, spotted fever group rickettsiae; STGO, scrub typhus group orientiae*.

Rickettsial diseases are military and public health concerns because they are distributed widely throughout the world (9–14). Though many rickettsial diseases are mild and self-limiting, there are several of them such as epidemic typhus, RMSF, scrub typhus, murine typhus, and MSF that can be quite severe and life threatening (6, 14). Such rickettsial agents have the potential for use as biological weapon (BW) agents (15). Since the early 2000s, the United States (US) Department of Defense (DoD) has funded and implemented a Biological Threat Reduction Program (BTRP) through the Defense Threat Reduction Agency (DTRA). DTRA BTRP funded multiple cooperative biological research (CBR) multi-year and Threat Agent Detection and Response Activity Project (TAP) single-year biosurveillance studies in countries throughout the world (16). Moreover, the Global Emerging Infections Surveillance (GEIS) Branch of the Armed Forces Health Surveillance Division, responsible for identifying military health relevant threats to inform force health protection decision making, has supported infectious disease surveillance globally (17, 18). These two agencies are major sources of funding supporting the development of and provision of rickettsial assays and methodology by the Rickettsial Diseases Research Program (RDRP) at the Naval Medical Research Center (NMRC). With this support, RDRP participates in international cooperative research and herein describes collaborations with nine countries resulting in the development/support of rickettsial disease research that provided partner countries with the capacity and capability to conduct rickettsial diseases surveillance that informed medical and scientific leaders as to the risk of rickettsial outbreaks in their area of responsibility.

The distribution of rickettsial diseases is varied throughout all continents except Antarctica (5–7). The specific knowledge of the presence, identity, prevalence, and distribution of the rickettsioses and their causative rickettsial agents are only partially known and varies significantly from country to country. This lack of knowledge is often directly tied to limited laboratory diagnostic capability and access to rickettsial assays and therefore places many countries and regions at risk of underestimating the impact and risk of rickettsial diseases, both sporadic occurrences and outbreaks (6). To overcome the shortfall of rickettsiology in underserved countries/regions, we have conducted cooperative research to determine the risk of various rickettsial diseases. Our team provided the rickettsial reagents and assays needed to initiate this work and increased local laboratory capability and capacity through general and specific laboratory training, access to and training on rickettsial assays and reagents, assistance with the evaluation of results, and drawing proper conclusions to be shared with local public health leaders and the international scientific community. The cooperative research among participating institutions has led to enhanced laboratory capability and reinforced knowledge on the presence, identity, distribution, and prevalence of rickettsial agents and diseases within their sphere of responsibility. This enhanced capability has led to partner country scientists' capacity to determine the risk of rickettsial diseases, identify outbreaks, publish results for general observation, and submission of grant applications to further rickettsial disease research (16, 19).

The goal of this paper is to describe the particular components utilized and outcomes obtained during the development of international cooperative rickettsial diseases research to determine the risk of rickettsial disease outbreaks in nine countries from 2008 to 2020: Azerbaijan, Chile, Georgia, India, Kazakhstan, Madagascar, Thailand, Ukraine, and Vietnam. The narrative is divided into specific areas that address the (1) development of collaborations; (2) general description of 17 research institutes from nine countries; (3) overall goals of the research projects for each institute or combination of institutions; (4) serologic and molecular assays utilized to assess the presence, identity, distribution, and prevalence of rickettsial agents; (5) risk assessments made for rickettsial diseases; (6) training provided; and (7) results obtained by partner countries and the important knowledge gain from the studies making great contribution to rickettsiology, and enhancement of capacity and capability of the institutes. Lastly, there is a discussion of the importance of continuing these collaborations, especially in regard to the results obtained. The overall benefit of international collaborations is to improve partner countries assessment of infectious diseases by enhancing their ability to accurately assess the risk of endemic diseases and the potential of outbreaks of infectious diseases such as the recent COVID-19 pandemic.

## Development of Collaborations

Discussions with potential collaborators were initiated in-person at conferences or correspondence by email, teleconference, through colleague referrals, and funding agencies' annual meetings. The Research Topics addressed included specific rickettsial disease research (*n* = 15 projects) as well as rickettsial disease research included in febrile disease projects (*n* = 2) and arthropod-borne and/or zoonotic disease research projects (*n* = 9). The proposals for these projects were initiated by collaborators with mutual interests but were often augmented by additional collaborators and institutions to broaden the scope. The final proposals were subsequently submitted to institutions for approval prior to submission to funding agencies. The institutes received approval for research grants from one or more funding organizations. The projects that were performed and their funding sources are shown in Table 2. The funding organizations included DTRA (*n* = 16), GEIS (*n* = 9), Nacional de Desarrollo Cientifico y Technologico (FONDECYT) (*n* = 7), National Foundation for Science and Technology Development of Vietnam (NAFOSTED) (*n* = 3), Indian Council for Medical Research (ICMR) (*n* = 3), Institut Pasteur de Madagascar (IPM) (*n* = 1), and Khon Kaen University (KKU) (*n* = 1).

**Table 2:** Collaborative rickettsial research projects by country.

**Host countries and institutes**	Project nomenclature^*^	**Projects**
**Azerbaijan** - Republican Antiplague Station, Baku; - Ministry of Defense, Head Medical Office, Baku; - Republican Hygiene and Epidemiology Center, Baku	DTRA AJ-TAP-2	A seroprevalence study of prior exposure to select arthropod-borne and zoonotic infections among rural populations in three regions of Azerbaijan.
	DTRA AJ-TAP-4	A prospective cohort study of the incidence and prevalence of select arthropod-borne and zoonotic infections among Azerbaijani military personnel.
	DTRA-RDRP	Analysis of tick samples from Georgia and Azerbaijan.
**Chile** - School of Medicine, Pontificia Universidad Católica de Chile, Santiago; - Facultad de Ciencias Veterinarias, Universidad Austral de Chile, Valdivia; - Clínica Alemana de Santiago Facultad de Medicina Clínica Alemana, Universidad del Desarrollo, Santiago	FONDECYT, OSU, and GEIS-RDRP	Potential scrub typhus case in Chile.
	FONDECYT and GEIS-RDRP	Assessment of domestic dogs for evidence of rickettsial infection.Case report of Korean traveler with scrub typhus.Distribution of scrub typhus cases in Chile.Identify potential scrub typhus vectors.Genetically characterize orientiae from scrub typhus cases.Conduct serosurvey of rickettsial disease in Chile.
**Georgia** - National Center for Diseases Control and Prevention, Tbilisi; - Laboratory of the Ministry of Agriculture, Tbilisi	GEIS-RDRP and DTRA:GG-21	Human disease epidemiology and surveillance of especially dangerous pathogens in Georgia.
	DTRA: GG-TAP-4 and DTRA: GG-TAP-12	Prevalence of *Rickettsia, Ehrlichia*, and *Borrelia* species pathogens in ticks from Georgia.Analysis of previously identified *Rickettsia-*positive Georgia ticks by multi-locus sequence typing.
**India** - Regional Medical Research Centre, Dibrugarh	DTRA-RDRP and ICMR	Assess individuals from Northeast India for seroprevalence of rickettsioses.Determine genetic characterization of *Orientia tsutsugamushi* in NE IndiaDetermine the risk of spotted fever in Northeast India.Determine the seroprevalence of typhus group rickettsia in Northeast India.
**Kazakhstan** - Uralsk Anti-plague Station, Uralsk; - Scientific Center of Quarantine and Zoonotic Diseases, Almaty - Scientific and Practical Center of Sanitary and Epidemiological Expertise and Monitoring, Almaty	DTRA: KZ-TAP-2	Species identification of tick vectors associated with infectious disease in Kazakhstan.
	DTRA: KZ-29	The epidemiology of Crimean-Congo hemorrhagic fever, hantavirus (hemorrhagic fever with renal syndrome), and tick-borne viral and rickettsial diseases in the Republic of Kazakhstan.
	DTRA: CAP-1 and DTRA: KZ-31	Flea-borne disease surveillanceandEffect of *Rickettsia* spp. upon fitness of *Yersinia pestis* in fleas that vector plague in the Republic of Kazakhstan.
**Madagascar** - Institut Pasteur de Madagascar, Antananarivo	IPM and GEIS-RDRP	Flea-borne rickettsial diseases in Madagascar.
**Thailand** - Khon Kaen University, Khon Kaen	GEIS-RDRP and KKU	Role of companion cats and cat fleas play in rickettsial diseases in Northeast Thailand.
**Ukraine** - Lviv Scientific Research Institute of Epidemiology and Hygiene, Lviv	DTRA PDG for UP-1	Evaluation of arthropod-borne infections in Ukraine.
**Vietnam** - Hanoi Medical University, Hanoi - National Hospital for Tropical Diseases, Hanoi	NAFOSTED	The presence and prevalence of rickettsial infections among humans in northern Vietnam.
	DTRA-RDRP and NAFOSTED	Characterize clinical manifestations of rickettsial diseases and determine the applicability of molecular assays in rickettsial diagnosis.
	DTRA-RDRP and NAFOSTED	Determine the genetic makeup of *O. tsutsugamushi* causing scrub typhus in Vietnam.

* *Project Nomenclature: RDRP, Rickettsial Diseases Research Program of the Naval Medical Research Center; DTRA, Defense Threat Reduction Agency; TADR, Threat Agent Detection and Response of DTRA; TAP, TADR Activity Project of DTRA; GEIS, Global Emerging Infections Surveillance and Response Research Support; DTRA-RDPR, Support in conducting rickettsial diseases research in CBR and TADR projects, and providing reagents and training; GEIS-RDRP, Development, production, and supply of assays/reagents to support rickettsial disease surveillance by GEIS partners worldwide; OSU, Ohio State University; FONDECYT, Nacional de Desarrollo Científico y Tecnológico; ICMR, Indian Council for Medical Research; IPM, Institut Pasteur de Madagascar; KKU, Khon Kaen University; NAFOSTED, National Foundation for Science and Technology Development of Vietnam*.

## Research Institutes by Country

Seventeen research institutes from nine countries collaborated with the Rickettsial Diseases Research Program (RDRP) of the Naval Medical Research Center (NMRC), Silver Spring, Maryland, USA, including (1) **Azerbaijan**: Republican Antiplague Station, Baku; Republican Hygiene and Epidemiology Center, Baku; and Ministry of Defense (MoD) Laboratory, Baku; (2) **Chile**: School of Medicine, Pontifi via Universidad Católica de Chile, Santiago; Facultad de Ciencias Veterinarias, Universidad Austral de Chile, Valdivia; Clínica Alemana de Santiago, Facultad de Medicina Clínica Alemana, Universidad del Desarrollo, Santiago; (3) **Georgia**: the National Center for Disease Control and Public Health (NCDC); and Laboratory of the Ministry of Agriculture, Tbilisi, Georgia; (4) **India**: Northeast Regional Medical Research Centre (NRMR), Dibrugarh, India; (5) **Kazakhstan**: M. Aikimbayev's National Scientific Center for Especially Dangerous Infections (NSCEDI), formerly named Kazakh Scientific Center of Quarantine and Zoonotic Diseases (KSCQZD) and the Scientific Practical Center for Sanitary Epidemiological Expertise and Monitoring (SPC-SEEM), Almaty, and Uralsk Anti-Plague Station (UAPS), Uralsk; (6) **Madagascar**: Institut Pasteur de Madagascar (IPM), Antananarivo; (7) **Thailand**: Khon Kaen University (KKU), Khon Kaen, Thailand; (8) **Ukraine**: Lviv Scientific Research Institute of Epidemiology and Hygiene (LSRIEH), Lviv; and 9) **Vietnam**: Hanoi Medical University (HMU) and the National Hospital for Tropical Diseases (NHTD), Hanoi, Vietnam.

## Overall Goals of the Research Projects for Each Institute or Combination of Institutions

The research goals of a single institute was often to investigate a newly described or recently rediscovered rickettsial disease(s) and/or agent(s) in a particular region in these countries: (1) **India:** “Identify previously recognized scrub typhus as well as determine the presence of other rickettsial diseases and/or agents in Northeast India” by RMRC, Dibrugarh, India; (2) **Madagascar:** “Identify flea-borne rickettsial agents near the capital city” by Institut Pasteur, Antananarivo, Madagascar; (3) **Thailand:** “Assess the role of cats and cat fleas in presence of spotted fever group rickettsiae in Northeast Thailand” by Khon Kaen University, Khon Kaen, Thailand; and (4) **Ukraine:** “Ascertain whether typhus group rickettsiae are still present and whether spotted fever group rickettsiae are present by assessing the seroprevalence of the agents infecting humans residing in Lviv Oblast,” by the LSRIEH, Lviv, Ukraine.

Unlike the above single-institute studies, many of the projects discussed below involved multiple institutes within a country, because investigating the presence and distribution of rickettsial diseases and/or agents was often a similar goal of multiple institutes due to the collaborative nature of the projects performed within the countries (e.g., Azerbaijan, Chile, Georgia, Kazakhstan, and Vietnam). These institutes worked together or independently on the following research goals within the countries: (1) **Azerbaijan:** Initially, there were three goals to assess the risk of rickettsial diseases in Azerbaijan, which involved multiple institutes: (a) “Determine presence of TGR and SFGR infections by seroprevalence study of rural populations in 3 regions of Azerbaijan”; (b) “Determine incidence and prevalence of rickettsial infections among a cohort of military individuals”; and (c) “Ascertain whether arthropods from rodents contained rickettsial agents.” These studies conducted independently were conducted by the Republican Antiplague Station, Baku; Republican Hygiene and Epidemiology Center, Baku; and Ministry of Defense (MoD) Laboratory, Baku, Azerbaijan. (2) **Chile:** Scrub typhus for hundreds of years was thought to be only found in the Asia–Australia region. So, when an individual presented to a clinic in 2006 in Chile with signs and symptoms of rickettsial disease, it was quite unexpected and even more unexpected that it was subsequently determined to be scrub typhus. This led to clinicians and researchers searching for further evidence of scrub typhus in Chile. The clinical and scientific investigations utilize various expertise of clinicians, scientists, and institutions. Thus, the overall goal in this country was to determine the clinical presentation, distribution, prevalence, incidence, vectors, reservoirs, and genetic characteristics of the disease and its agents. Thus, the multiple institutions worked well together on various aspects of the overall goal. The institutes included the following: School of Medicine, Pontifi via Universidad Católica de Chile, Santiago; Facultad de Ciencias Veterinarias, Universidad Austral de Chile, Valdivia; Clínica Alemana de Santiago, Facultad de Medicina Clínica Alemana, Universidad del Desarrollo, Santiago, Chile. (3) **Georgia:** For the country of Georgia, a current assessment of rickettsial diseases/agents was needed as only limited knowledge of rickettsial diseases existed. Thus, the goal was to ascertain the presence of rickettsial infections and rickettsial agents in Georgia by (a) determining the role of rickettsial agents among febrile patients and (b) assessing ticks for the presence of rickettsial agents and specifically identifying them with new molecular assays. The institutes involved in these studies included the National Center for Disease Control and Public Health (NCDC); and Laboratory of the Ministry of Agriculture, Tbilisi, Georgia. (4) **Kazakhstan:** Tick-borne (Crimean-Congo hemorrhagic fever; tick-borne encephalitis) and flea-borne (plague) diseases are endemic to Kazakhstan. However, there was only limited knowledge of the presence of rickettsial diseases in Kazakhstan. Thus, the overall goal of the rickettsial disease studies was to augment the minimal knowledge of the presence of rickettsiae in Kazakhstan. Thus, various studies of rickettsial disease agents were investigated, especially those associated with ticks and fleas, including (a) “Identify Tick-borne Rickettsial Agents in Kazakhstan” and (b) “Determine the Presence and Distribution of Flea-borne Rickettsiae in Kazakhstan” conducted by NSCEDI and SPC-SEEM Almaty, and UAPS Uralsk, Kazakhstan; and (5) **Vietnam:** Similar to other countries, Vietnam had historical evidence of rickettsial diseases; however, for several decades, there was limited investigation into the presence, prevalence, and distribution of rickettsial diseases and agents. Thus, the overall goal for Vietnam in the past decade has been to rectify the deficiency by conducting seroprevalence, clinical, and environmental studies in various number of locations. Three studies addressed the limited knowledge of rickettsial infections in northern Vietnam: (a) “The presence and prevalence of rickettsial infections among humans in northern Vietnam”; (b) “Characterization of the clinical manifestations of rickettsial diseases and determine the applicability of molecular assays in rickettsial diagnosis”; and (c) “Ascertain the genetic makeup of *Orientia tsutsugamushi* causing scrub typhus in northern Vietnam.” The institutes involved in these studies included the National Hospital for Tropical Diseases and the Hanoi Medical University, Hanoi, Vietnam.

## Laboratory Assays

### Serological Assays

Commercially available and NMRC's serological assays that they developed in-house and not for commercial use were utilized. NMRC's serological assays with standard operating procedures (SOPs) included typhus group rickettsiae (TGR)-specific enzyme-linked immunosorbent assay (ELISA)-immunoglobulin gamma (IgG), spotted fever group rickettsiae (SFGR)-specific ELISA-IgG, and scrub typhus group orientiae-specific (STGO) ELISA-IgG (13, 20, 21). Positive controls (*n* = 1) and negative controls (*n* = 3) for each assay were provided to confirm that the assays were performing correctly (4). Subsequently, the institutions identified positive and negative control sera from their studies to use in these assays.

Commercial serological assays with instructions for ELISA and indirect immune fluorescence assay (IFA) were used according to manufacturers' instructions. These assays included InBios Scrub Typhus IgM ELISA (InBios International Inc., Seattle, WA) and Scrub Typhus IFA-IgG (Fuller Laboratories, Fullerton, CA).

### Molecular Assays

#### PCR

Four types of polymerase chain reaction (PCR) were used: (1) standard PCR (sPCR), (2) nested PCR (nPCR) or hemi-nested PCR (hnPCR), and (3) quantitative real-time PCR (qPCR) assays.

#### qPCR

The qPCR assays (genus-, group-, or species-specific) were either developed at NMRC or found in peer-reviewed publications, and were used to screen for and identify rickettsial and oriental agents (4). The primers, probes, and controls [positive controls included either plasmids containing the target gene fragment(s) or linear target gene fragment(s), and molecular-grade water served as the negative controls] were used as described (4). Either reagents for the qPCR assays were supplied; their product numbers were provided; the primers, probes, and linear positive control oligonucleotide sequences were provided; or a combination of reagents, oligonucleotide sequences, and product numbers were provided. With the provided information for the reagents, the institutes could subsequently obtain the reagents independently of NMRC.

#### sPCR/nPCR

The sPCR and nPCR/hnPCR were used to produce amplicons for specific gene fragment sequencing either for a single gene or multiple genes in multilocus sequence typing (MLST) to identify and characterize rickettsial agents (4). Multiple gene fragment sequences were used as described in the MLST scheme initially described by Fournier et al. (22) to identify known *Rickettsia* species, incompletely characterized *Candidatus* Rickettsia species, and not previously described rickettsial agents (4). The genes most commonly used in the described studies for the identification of rickettsiae included 17-kDa antigen gene, *rrs, gltA, ompB, ompA*, and *sca4* (4). Identification of *Orientia* species by MLST utilized the following genes: *rrs*; 47-kDa antigen high-temperature requirement A protease gene (*htrA*); and the 56-kDa type-specific antigen gene (*tsa56*) (23).

#### Sequencing and Phylogenetic Analysis

The products from sPCR/nPCR for a single gene or for multiple genes to conduct MLST were sequenced in-house or using commercial companies as previously described (24). The sequencing data were assembled by Lasergene version 15.0 software (DNASTAR, Inc. Madison, WI, USA) or similar software, and sequences were compared with sequences available in GenBank (NCBI) using the BLAST search tool (https://blast.ncbi.nlm.nih.gov/Blast.cgi?PAGE_TYPE=BlastSearch).

Gene sequences, excluding the primer regions, were aligned by the ClustalW and phylogenetic analysis performed using MEGA X software (or similar software). The phylogenetic trees were constructed based on the alignment of the various gene fragment sequences (described above) obtained using the maximum likelihood method and Tamura-Nei model (25), and bootstrap analysis (1,000 reiterations) was carried out according to the Kimura 2-parameter method. All positions containing alignment gaps and missing data were eliminated.

## Risk Assessments

Assessing the risk of endemic rickettsial diseases and the potential for outbreaks requires measuring epidemiological metrics such as determining the presence, spatial and temporal distribution, prevalence for individual samples or minimal infection rate for pooled samples, and incidence of rickettsial infections and their causative agents. The evidence of rickettsial infections (e.g., antibodies against group-specific rickettsial antigens and/or detection of rickettsial agents in clinical samples) clearly indicates the presence of rickettsial pathogens in a location/region/country. Subsequent studies to determine their prevalence, incidence, and distribution are required to better localize the risk of rickettsioses. Molecular studies utilizing assays such as qPCR provide the specificity required to identify *Rickettsia* pathogens; however, if only genus- or group-specific assays are used, non-pathogens within the genus or group may be detected and confused with pathogens. Therefore, more species-specific assays are needed in accurately determining rickettsial disease risk assessments.

## Training

NMRC staff scientists trained and mentored partner country scientists involved in the rickettsial diseases research projects prior to and during performance of laboratory work (Table 3). Training provided was project driven and included the following subjects: (1) conducting BSL-2 general laboratory procedures as they pertained to rickettsial studies; and (2) developing, updating, and/or augmenting SOPs to include rickettsial specific assays and general procedures for sequencing and MLST. New assays/procedures utilized in the laboratories were conducted with appropriate controls and standards to evaluate performance. Once partner country scientists were confident with the new procedures, they used the assays to assess environmental and/or clinical samples. Performance of laboratory assays was routinely assessed by comparing results of controls with the same controls performed at NMRC or described by manufacturers. The commercial and in-house assays and their sources that were utilized by the various laboratories are shown by country in Table 4.

**Table 3:** Training utilized by research institutes to identify rickettsiae and rickettsial infections.

**Training**	**Development of a protocol(s) to assess risk or rickettsial diseases utilizing rickettsial laboratory procedures utilizing pre-existing and/or collection of environmental and/or clinical samples**	**Rickettsiology and introduction to laboratory assays for obtaining evidence of rickettsial diseases and agents**	**SOPs: review/updated for general procedures of BSL-2 laboratories**	**Laboratory assays: general, serological, and /or molecular**	**Use of commercial and non-commercial serologic assays to detect evidence of previous rickettsial infection—group-specific**	**Use of commercial and non-commercial molecular assays to detect and identify rickettsiae**	**Determine identity of rickettsiae by sPCR, nPCR, and MLST using GFS and phylogeny**
Azerbaijan	AJ-TAP-2; AJ-TAP-4	Yes	BSL-2	Yes, yes, yes	Non-commercial ELISA TGR and SFGR	Non-commercial Rick17b and RfelG qPCR assays	n/a
Chile	FONDECYT-need title and GEIS-RDRP	Yes	BSL-2	Not needed	Commercial and non-commercial: TGR, SFGR, STGO	Non-commercial: Otsu47; Orien16S	Yes for *rrs, htrA*, and *tsa56*
Georgia	GG-TAP-4; GG-TAP-12; GG-21	Yes	BSL-2	Not needed	Non-commercial ELISA TGR, SFGR and STGO	Non-commercial: Rick17b, Raesch, Rraoul, Rslov, Rmon, Rconor, and Rmass9666 qPCR assays	Yes for *gltA, ompA, ompB*, and *sca4*
India	ICMR-need title and DTRA-RDRP	Yes	BSL-2	Yes, yes, no	Commercial and non-commercial STGO, TGR and SFGR ELISAs	n/a	n/a
Kazakhstan	KZ-29; KZ-31; KZ-TAP-2; KZ-CAP-1	Yes	BSL-2	Yes, yes, yes	Non-commercial ELISAs for TGR and SFGR	Non-commercial Rick17, RfelB, Rasemb	n/a
Madagascar	IP-need title and GEIS-RDRP	Yes		Yes, yes, yes	Non-commercial STGO, TGR and SFGR ELISAs	Non-commercial Rtyph and RfelB	n/a
Thailand	KKU-need title; GEIS-RDRP	Yes		Yes, yes, yes	Non-commercial ELISAs for SFGR	Non-commercial Rick17b.	n/a
Ukraine	UP1-UDP	Yes	BSL-2	Yes, yes, no	Non-commercial ELISAs for TGR and SFGR	n/a	n/a
Vietnam	DTRA-RDRP and NAFOSTED	Yes	BSL-2	Yes for new ELISAs and qPCR assays	Commercial and non-commercial: TGR, SFGR, STGO	Non-commercial: Rick17b, Rtyph and Otsu47 qPCR assays	Yes for *tsa56*

**Table 4:** Serological and molecular assays utilized in rickettsial investigations by country.

**Countries**	Source of assays^*^	**Serologic evidence of rickettsial infections (group-specific assays) Molecular evidence of rickettsiae (genus-, group-, and/or species-specific assays)**		
		Type of assays^**^	Specific assays^***^	Host samples^****^
Azerbaijan	NMRC	ELISAs	TGR- and SFGR-ELISAs-IgG	Human sera
		qPCR	Rick17, Trick, RfelG, Raesch, Rraoul, Rslov	Tick
Chile	NMRC	ELISAs	STGO	Human serum
	OSU	Sequencing	*rrs*	Human eschar
	NMRC	ELISA	STGO	Dog sera
	InBios Fuller Laboratories	ELISA-IgMIFA-IgG	*O. tsutsugamushi* antigens	Human serum
	NMRC	qPCR	Otsu47	Human eschar
		MLST	*rrs, htrA*, and *tsa56*	Human eschar
	InBios Fuller Laboratories	ELISA-IgM and IgGIFA-IgG	*O. tsutsugamushi* antigens	Human sera
	NMRC	qPCR	Orien16S	Human eschas
		MLST	*rrs* and *htrA*	
	NMRC	qPCR	Orien16S	Mites
	NMRC	qPCR	Orien16S	Human eschars and buffy coat
	PUCC	MLST	*rrs, htrA*, and *tsa56*	
	NMRC	ELISAs	TGR-, SFGR-, and STGO-ELISAs-IgG	Human sera
Georgia	NMRC	qPCR assays	Rick17, Trick, Raesch, Rraoul, Rslov	Ticks
		MLST	*gltA, ompA, ompB, sca4*	
		ELISAs	TGR- and SFGR-ELISAs-IgG	Human sera
		qPCR assays	Rick17b, Rraoul, Rslov, Raesch, Rmona, Rconor, Rmass9666	Ticks
		MLST	*gltA, ompA, ompB, sca4*	
India	NMRC	ELISAs	TGR-, SFGR-, and STGO-ELISAs-IgG	Human sera
	InBios	ELISA	InBios Scrub typhus ELISA-IgM	
	NMRC	Sequencing	*tsa56*	Human blood
		ELISAs	TGR-, SFGR-, and STGO-ELISAs-IgG	Human sera
		MLST	17kDa, *gltA, ompA, ompB, sca4*	Human blood
		ELISA	TGR-ELISA-IgG	Human sera
Kazakhstan	USAMRIID	qPCR assays	*Dermacentor, Hyalommma, Rhipicephalus*, and *Ixodes* genus-specific assays	Ticks
	NMRC	qPCR	Rick17b	Ticks
		qPCR	Rick17b	Ticks
		qPCR assays	Rick17b, Raesch, Rraoul, Rslov	Ticks
		qPCR assays	Rick17b, Rtyph, RfelG, RfelB, Rasemb	Rodent fleas
Madagascar	NMRC	ELISAs	TGR- and SFGR-ELISAs-IgG	Human seraSmall mammal sera
	NMRC and IPM	qPCR assays	RKND, RfelG, Rasemb	Small mammal fles
	NMRC and IPM	Sequencing	*ompB*	
Thailand	NMRC	ELISA	SFGR-ELISA-IgG	Cats
		qPCR assays	Rick17b, Rtyph, RfelG, RfelB, Rasemb	Cat fleas
		Sequencing	*ompB*	
Ukraine	NMRC	ELISA	TGR-ELISA-IgG	Human
			SFGR-ELISA-IgG	
Vietnam	NMRC	ELISAs	TGR-, SFGR-, and STGO-ELISAs-IgG	Human sera
		ELISAs	TGR-, SFGR-, and STGO-ELISAs-IgG	Human sera
		qPCR assays	Otsu47, Rick17b, Rtyph	Human buffy coats
		qPCR	Otsu47	Human PBMCs
				Human eschars
	HMU and NHTD	Sequencing	*tsa56*	Human PBMCs
				Human eschars

* *Source of assays: NMRC, Naval Medical Research Center; OSU, Ohio State University; InBios, InBios International, Inc; USAMRIID, United States Army Medical Research Institute of Infectious Diseases; IPM, Institut Pasteur de Madagascar; HMU, Hanoi Medical University; NHTD, National Hospital for Tropical Diseases*.

** *Type of assays: ELISAs, enzyme-linked immunosorbent assays; qPCR, quantitative real-time PCR; MLST, multilocus sequence typing*.

*** *Specific assays: TGR, typhus group rickettsiae; SFGR, spotted fever group rickettsiae; STGO, scrub typhus group orientiae; IgG, immunoglobulin gamma; Rick17, a genus-specific qPCR assay for Rickettsia species (1st generation); Rick17b, a genus-specific qPCR assay for Rickettsia species (2nd generation); RKND, a genus-specific qPCR assay for Rickettsia species; Trick, a group-specific qPCR assay for tick-borne spotted fever group Rickettsia species; Rtyph, a species-specific qPCR assay for Rickettsia typhi; Rasemb, a species-specific qPCR assay for Rickettsia asembonensis; RfelG, a group-specific qPCR assay for flea-borne spotted fever group Rickettsia species; Orien16S, a genus-specific qPCR assay for Orientia species; Raesch, a species-specific qPCR assay for Rickettsia aeschlimannii; Rraoul, a species-specific qPCR assay for Rickettsia raoultii; Rslov, a species-specific qPCR assay for Rickettsia slovaca; Rmona, a species-specific qPCR assay for Rickettsia monacensis; Rconor, a subspecies-specific qPCR assay for Rickettsia conorii subsp. conorii; Rmass, a species-specific qPCR assay for Rickettsia massiliae; rrs, 16S rRNA gene; htrA, 47-kDa antigen gene; tsa56, 56-kDa type-specific antigen gene; 17 kDa, 17-kDa antigen gene; gltA, citrate synthase gene; ompA, outer membrane protein A gene; ompB, outer membrane protein B gene; sca4, surface cell antigen 4 gene*.

**** *Host samples: PBMC, peripheral blood mononuclear cell*.

## Specific Findings by Country

The results of the various rickettsial disease research projects (*n* = 26) were completed at 17 institutions from nine countries and are described in detail below by country. The findings are summarized and appropriate references provided in Table 2 and Supplementary Table 1.

### Azerbaijan

Serological data showed the prevalence of IgG against SFGR (3.7–15.9%) and TGR (0–0.6%) among individuals in Azerbaijan and indicated a low to moderate exposure to SFGR and a very low risk of TGR infections (Supplementary Table 1). Antibodies against SFGR are lifelong and therefore the point prevalence studies do not allow for a strong evaluation of outbreak risk. Additional serosurveys throughout Azerbaijan need to be performed, as well as tick and flea field studies in locations positive and negative for evidence of rickettsia agents following results of serosurveys. Moreover, clinical studies should be carried out to assess incidence over time as well as during outbreaks to determine the risk levels of endemic rickettsioses spatially and temporally. Molecular evidence of *R. felis* group was found among a small number of ixodid ticks collected from rodents in the Lankaran district located in the southeast of Azerbaijan near the border with Iran (Supplementary Table 1). However, the total number of arthropods collected was low, and the assay used to identify *R. felis* in this study has subsequently been found to have low specificity, both of which decrease our ability to determine risk of human infection with *R. felis* group of rickettsiae in this district or Azerbaijan. More arthropod surveys, both ticks and fleas, are needed.

### Chile

The first recognized human case of scrub typhus in 2006 in South America was confirmed by molecular and serological studies and reported in 2011 (26) (Supplementary Table 1). An additional three cases were subsequently identified and reported in 2016 (27). Since then, more than 40 cases have occurred throughout Chile (Supplementary Table 1). The disease has been characterized clinically, and serological studies of dogs (sentinel animals) and humans have shown the distribution both locally and nationally (Supplementary Table 1). In addition, human serological studies have identified spotted fever and typhus group rickettsial infections in Chile. Molecular characterization of the causative agent, *Candidatus* Orientia chiloensis, from human, rodent, and mite samples has shown that it is distinct from *O. tsutsugamushi* and *Candidatus* O. chuto (Supplementary Table 1). Additional studies are needed to show the transmission of the agent by mites to confirm the vector and hospital studies to better characterize the disease, incidence, and potential for scrub typhus outbreaks in Chile.

### Georgia

Initially, only a single rickettsial agent, *Rickettsia conorii*, was known to be endemic to Georgia (28). More recently, ixodid ticks acquired from livestock, trapped rodents, and tick drags in May 2008 (*n* = 653) and 2009 (*n* = 264) from areas in the districts of Akhaltsikhe, Aspindza, Gori, and Kaspi of the country of Georgia were found to contain three additional rickettsial pathogens *R. aeschlimannii, R. roultii*, and *R. slovaca* (Supplementary Table 1). Additional studies followed utilizing new molecular assays and MLST to characterize the rickettsiae that were unable to be identified by qPCR. In the end, a total of nine tick-borne rickettsiae, six pathogens, and three rickettsial agents of unknown pathogenicity were identified. A hospital-based fever study determined that among 655 fever patients, 10 (1.5%), 2 (0.3%), and 2 (0.3%) patients had evidence of a previous infection with SFGR, STGO, and TGR, respectively (Supplementary Table 1). These results suggest the presence of rickettsial diseases among Georgians but at a very low prevalence, suggesting that the risk of a rickettsial outbreak is low. These results need to be confirmed with additional nationwide hospital-based fever studies.

### Northeast India

Four epidemiological studies of rickettsial infections were initiated after it was discovered that scrub typhus had returned to Northeast India after a gap of 67 years (29). In the first study, of 1,264 human serum samples assessed by ELISA-IgG 390 (30.8%), 175 (13.8%), and 53 (4.2%) individuals had antibodies against STGO, SFGR, and TGR, respectively. Molecular studies of the positive serum samples identified two individuals had *O. tsutsugamushi* DNA. Investigation of arthropods (ticks, fleas, and mites) from domestic animals and rodents of the study area found only fleas that were positive for rickettsiae by the Rick17b genus-specific qPCR assay (4 of 16 individuals) and sequencing determined that the agent was *Candidatus* R. senegalensis (Supplementary Table 1). This study showed conclusively the presence of rickettsial diseases in this underserved area of India. In the second study, to determine the genotype of *O. tsutsugamushi* causing scrub typhus in NE India, patients screened positive by InBios Scrub Typhus ELISA-IgM blood samples were assessed for the presence of *tsa56*. Those positive amplicons were sequenced and three distinct genotypes were identified (Supplementary Table 1). This knowledge can be used in planning control strategies and prophylactic measures and identifying outbreak sources. The third study, involving the investigation of serum samples (*n* = 317) from individuals in another scrub typhus endemic region of NE India, found seroprevalence against scrub typhus, typhus, and spotted fever of 35.6, 2.2, and 0%, respectively. DNA extraction of seven SFGR positive blood samples only obtained a single amplicon for the 17-kDa gene. Sequence typing identified *R. felis*, a flea-borne rickettsia associated worldwide with flea-borne spotted fever (Supplementary Table 1). Future studies are needed to look for *R. felis* infection in patients with fever of unidentified origin (FUO) to determine the epidemiology and to understand the complex paradigm of *R. felis* transmission in India. Lastly, a study involving 2,199 clinical patients (762 with acute encephalitis syndrome and 1,437 with FUO) and 40 (1.8%) samples were found positive for IgG against *R. typhi* (Supplementary Table 1). This prevalence among patients suggests that a low risk of murine typhus exists in NE India and thus additional epidemiological studies throughout NE India should be performed to assess the risk of murine typhus with special attention to urban settings.

### Kazakhstan

To assess arthropod-borne rickettsial diseases in Kazakhstan, new molecular methods were introduced to identify tick and *Rickettsia* species via United States Army Medical Research Institute of Infectious Diseases and NMRC in collaboration with NSCEDI-KSCQZD, SPC-SEEM, and UAPS. In Kazakhstan, North Asian tick typhus, also known as Siberian tick typhus, caused by *Rickettsia sibirica*, is endemic. However, it was unclear if other tick-borne diseases due to rickettsial infections occurred. To address this issue, *Rickettsia* species were identified initially only at the genus level with the Rick17b qPCR assay. The preliminary results showed the presence of tick-borne rickettsiae in Kazakhstan and the utility of molecular assays in arthropod-borne rickettsial surveillance. The addition of three species-specific qPCR assays identified never before known endemic regions for *R. aeschlimannii, R. raoultii*, and *R. slovaca*. In addition, flea-borne rickettsiae were identified in southwestern Kazakhstan that included *R. felis/Ca*. R. senegalensis and *R. asembonensis* (Supplementary Table 1). These new rickettsial pathogens identified in Northern, Western, Southern, and Southwestern Kazakhstan were significant in identifying health issues and stimulation of additional surveillance studies and brought awareness of the potential for rickettsial outbreaks in addition to the recognition of potential endemic rickettsioses.

### Madagascar

Due to the presence of flea-borne diseases such as plague in Madagascar, this study was conducted to determine if a risk to flea-borne rickettsial diseases also exists in Madagascar. Notably, a vector for both plague and murine typhus is the Oriental rat flea (*Xenopsylla cheopis*). Thus, a survey among humans and rodents was conducted to assess the risk of flea-borne rickettsial diseases. A seroprevalence study was performed among humans and peri-domestic small mammals. The seroprevalence of SFGR and TGR among humans was 34 and 39%, respectively. However, among the small mammals collected, only 4.4% were IgG positive against TGR *R. typhi* antigens and none of the animals had evidence of IgG against SFGR antigens. Interestingly, among the peri-domestic small mammals' fleas collected and assessed for rickettsiae, 24.3 and 1.9% of Oriental rat fleas (*X. cheopis*) were positive for *R. typhi* (a TGR) and *R. felis* (a SFGR), respectively, and 30.8% of *P. irritans* were positive for *R. felis* (Supplementary Table 1). These results showed that at least the two rickettsial agents identified in the rodent fleas assessed have possible roles in the TGR and SFGR infections among people in Madagascar. Additional studies to determine the spatial and temporal distribution of flea-borne rickettsiae are needed, in addition to hospital-based studies to determine incidence of these rickettsial diseases.

### Northeast Thailand

A study of the role of domestic cats in the presence of rickettsial disease in Northeast Thailand investigated cat sera (42 serum samples) for the presence of antibodies against SFGR and cat fleas (*n* = 23) for molecular evidence of rickettsiae. Two cats (4.8%) had antibodies against SFGR, and 21 cat fleas (91.3%) were positive for *R. asembonensis* DNA (Supplementary Table 1). Thus, for Northeast Thailand, cats and cat fleas show evidence of spotted fever group rickettsiae, and physicians and veterinarians should be aware of the risk for rickettsial disease. These preliminary results should be followed up with additional surveillance and hospital studies to more clearly determine the risk of rickettsial diseases among the animal and human populations in Thailand.

### Ukraine

Seroprevalence of 1,000 non-febrile hospital patients in western Ukraine for TGR was determined to be 1.5%, and for SFGR, it was 5.1%, indicating a low prevalence among non-febrile patients from two hospitals for previous exposure to rickettsiae (Supplementary Table 1). Remarkably, seropositivity to TGR was only 1.5%, and the study population included people who lived in an area where epidemic typhus was previously endemic. The seroprevalence studies should be increased to take in all of Ukraine. Moreover, a hospital-based fever study should be conducted to assess the identities, incidence, and distribution of rickettsial diseases for Ukraine.

### Vietnam

Historically, rickettsial disease has been understudied in Vietnam. The following three studies conducted in northern Vietnam have added significantly to the knowledge of the presence of various rickettsial diseases, including scrub typhus and murine typhus and potentially spotted fever. In a serological study, the seroprevalence was determined to be 6.5, 1.1, and 1.7% for TGR, SFGR, and STGO infections of healthy humans, indicating the presence of all three groups infecting people in northern Vietnam, but at low levels. A subsequent study of hospitalized patients at two referral hospitals in Hanoi found that 34.1 and 3.3% of patients suspected of rickettsial disease were confirmed by serological and molecular assays to have scrub typhus and murine typhus, respectively. To follow up on these results, a second hospital-based study was conducted to characterize the *O. tsutsuagmushi* causing scrub typhus in northern Vietnam. It was determined that three geno-groups (Karp, Kato, and Gilliam) predominated. This information is important in the development of laboratory assays and vaccine candidates. Studies on rickettsial diseases with an emphasis on scrub typhus and murine typhus, but also including spotted fever, should continue to precisely determine the risk of endemic disease and the possibility of outbreaks for all of Vietnam.

In addition to providing leaders of the partner countries, research institutions, funding organizations, scientists, and clinicians with important rickettsial disease surveillance information based on these findings, the research was also made available to the international scientific community by providing abstracts to presentations (*n* = 40) given at national and international conferences and the publication of peer-reviewed articles (*n* = 18) in international scientific journals (Table 5). The important contributions of the rickettsia surveillance conducted due to the collaborations described herein to the scientific community for each country is exemplified when comparing the number of publications of rickettsial research before and after the collaborations. By searching PubMed for rickettsia, and rickettsia associated with arthropod-borne diseases and zoonosis, we found the number of publications varied significantly by country, with the most articles (without counting those described herein) published by India (281), Thailand (165), Chile (18), Vietnam (12), Ukraine (5), Madagascar (5), and Kazakhstan (3). Azerbaijan and Georgia had no other rickettsia publications by the PubMed search within the last 10 years other than the two described herein (Table 5). Thus, with the exception of India and Thailand, the percentage of publications due to those reported herein from seven countries (mean, 47.6%; range, 0–100%) shows the important knowledge that collaborative research provides to the partner institutes and global health.

**Table 5:** Rickettsia abstracts and publications by country.

**Countries**	**Number of rickettsia abstracts due to collaborations (reference numbers)**	**Number of rickettsia publications due to collaborations (reference numbers)**	Number of rickettsia publications from PubMed Search^***^ (references numbers)	**Number of rickettsia publications by collaboration/ total publications in past 10 years**
Azerbaijan	4 (30–33)	1 (34)	0	1/1 (100%)
Chile	8 (35–42)	5 (43–47)	19 (27, 48–65)	5/24 (20.8%)
Georgia	12 (66–77)	2 (34, 78)	0	2/2 (100%)
Northeast India^*^	2 (79, 80)	2 (81, 82)	4 (29, 83–85)	2/6 (33.3%)
Kazakhstan	8 (86–93)	3 (16, 19, 94)	1 (95)	3/4 (75%)
Madagascar	0	1 (96)	5 (97–101)	1/6 (16.7%)
Northeast Thailand^**^	1 (102)	1 (103)	2 (104, 105)	1/3 (33.3%)
Ukraine	2 (106, 107)	0	5 (108–112)	0/5 (0%)
Vietnam	3 (113–115)	3 (116–118)	12 (119–130)	3/15 (20%)

* *Search results for only Northeast India. Search results for all of India were 281 items*.

** *Search results for only Northeast Thailand. Search results for all of Thailand were 165 items*.

*** *Search words: rickettsia, arthropod borne, and zoonosis; search time period: past 10 years—does not include those listed for collaborations*.

## Discussion

During the implementation of our cooperative research, each country and institute(s) faced similar and unique challenges. Some laboratories were able to quickly incorporate rickettsial disease research or augment what they had into a self-sustaining capability. The primary challenge shared by all of the laboratories was recognizing the limited knowledge on rickettsial disease presence, identity, distribution, and prevalence, within their area of responsibility. Moreover, clinical presentations of rickettsial diseases are not distinct and are easily confused with multiple other infectious diseases such as dengue, leptospirosis, flu, malaria, etc. (6, 14). This issue is compounded by the lack of access to reliable diagnostic tests, especially in resource-limited and middle-income countries (2). Certain issues could not be overcome, especially those political in nature that arose between collaborating countries.

NMRC staff scientists provided training as needed and scheduled to conduct serological and molecular assays utilized to identify rickettsia and rickettsial infections (4). Performance of general laboratory procedures was discussed early on to ensure that all involved had the same knowledge and perceptions of working in BSL-2 laboratories. Trainings for rickettsial assays were accomplished in a mentoring fashion that included didactic lectures, hands-on laboratory instruction, and overseeing supervised instruction. Positive and negative controls, initially provided by NMRC, were utilized to ensure that laboratory results were obtained similarly in the partner laboratory and NMRC laboratory. Follow-up training was conducted as new procedures were added to the institutes' portfolios. This additional training was performed in the partner countries during visits and at NMRC where scientists from Chile, Georgia, India, Kazakhstan, and Vietnam visited and worked side by side with members of RDRP.

NMRC staff scientists also provided guidance and training on data analysis especially in the proper evaluation of laboratory results, most importantly, data quality: when to accept and when not to accept the results. This required the evaluation of standard controls (commercial and non-commercial) as acceptable ranges established and could be used for reference and quality control. Understanding how to analyze results relative to the controls and to know when there is an issue with a control are vital for interpreting results. This allowed for the development of appropriate conclusions from data obtained (4, 131–133). Examples of these discussions often occurred during partner country visits, side meetings of conferences, and video conference calls.

When detection of rickettsial disease is limited to serological data, it is difficult to determine the causative agent responsible for antibody response in a patient. Thus, the knowledge necessary to assess the specific rickettsial risk(s) is relegated to the general assumptions as to the cause of the infection, being limited to group specificity. Therefore, it is important to obtain specific information associated with proposed agents such as the host(s), the hosts' natural settings, prevalence of infected hosts, and their distribution (4, 14, 133).

Limited molecular identification of rickettsiae to the genus or group level in the environment does not allow one to determine accurately the risk of particular rickettsial diseases. Detection of pathogenic rickettsiae must be accomplished by *Rickettsia* species identification, as sympatric non-pathogenic rickettsiae can be found among the same arthropod hosts (134). For example, one can commonly find sympatric pathogens with non-pathogenic *Rickettsia* species among the same arthropod species [e.g., *Rickettsia rickettsii* and *Rickettsia montanensis* in *Dermacentor variabilis* (135); *Rickettsia parkeri* and *Rickettsia andeanae* in *Amblyomma maculatum* (136, 137); *Rickettsia felis* and *Rickettsia asembonensis* in *Ctenocephalides felis* (24, 138)].

The commonly used method of preparing arthropod vectors is to employ pooling, which groups the same arthropod species from one location, the same host, or drag/flag sheet into one pool sample (94). The pool samples can be restricted to individuals of the same arthropod species and life stage. As indicated above, since more than one rickettsial agent may be found within a single species of tick or flea, then pooled samples may contain more than one *Rickettsia* species. In this situation, one of the *Rickettsia* species might be missed and therefore one cannot determine the prevalence of each rickettsia accurately. The minimum infection rate (MIR) equals the total number of positive pools divided by the total number of individual arthropods in all the pools assessed multiplied by 100. A positive pool with 5 individuals in it is not counted the same as a pool with 50 individuals; i.e., the prevalence of each pool would be considered 100%, whereas the MIR would be considered to be 20 and 5%, respectively. If a pool has more than one *Rickettsia* species in it, then it will be assumed that only one agent of each species is among the pool sample (78). For example, if a pool of 10 *A. maculatum* individuals had both qPCR assays for *R. parkeri* and *R. andeanae* as positive, then the MIR for both agents would be 10%. Therefore, the most accurate way, though more time-consuming and costly, is to determine the prevalence of rickettsiae and the agents' identity within their arthropod hosts based on individual arthropods, not pools. Lastly, if you have both individual and pooled samples, you will have to determine both prevalence and MIR for the respective sample types (78). By definition, a pool sample cannot contain a single arthropod. To address the issues above, a strategy of processing arthropod hosts individually, screening by pools, and testing each individual arthropod in a positive pool can be used.

Unlike the genus *Rickettsia* where there is much more information on the pathogenicity of the individual species, significantly less is known about *Orientia* species. Moreover, the rodent animal models for *O. tsutsugamushi* strains do not necessarily mimic the virulence of the same agents in humans and non-human primates, thus confounding the matter of identifying pathogens (139, 140). However, it has been known for a very long time that differences in pathogenicity exist among the large diversity of *O. tsutsugamushi* strains (141). The scrub typhus story becomes more compelling now that we know there are additional *Orientia* species found outside the endemic region of *O. tsutsugamushi* (known as the Tsutsugamushi Triangle incorporating lands in Asia, Australia, and islands in the Indian and Pacific oceans). Scrub typhus is now considered endemic for multiple areas throughout the world (7). With the recent added complexity of orientiae, there is need for more research to be performed to determine presence of pathogen and non-pathogens within *Orientia*. For the present, detection of *Orientia* species is considered evidence of a causative agent for and therefore used in the risk assessment of scrub typhus.

The projects described herein clearly demonstrate that the partner country laboratories benefited from enhanced training, capacity/capability, education (science, laboratory procedures, language, and risk assessment), and appropriate funding support. The ultimate consequences were not only the data collected, which, following analysis, allowed the institutes and partner countries to determine risk of rickettsial diseases for districts, regions, and/or countries and which can subsequently be used by medical leaders and policy makers to institute further education and projects and augment preventive medicine, diagnostic, and treatment modalities to enhance public health. The true success of these projects is best measured by the ability of the partner laboratories to continue research on their own in this area of research as well as research in other infectious diseases. Fortunately, as can be seen by this paper and others (16, 19), the collaborating governments, funding partners, and research institution involvements were extremely positive, resulting in host country institutions and personnel enhancement in conducting rickettsial disease research, which also enhanced scientific and medical knowledge worldwide. Ultimately, collaborative researchers are able to provide information to public health decision makers with advanced awareness on emerging infectious disease threats such as rickettsial diseases, and thereby promote the timely, science-based disease outbreak prevention, preparedness, and control-and-response actions necessary to improve global public health.

## Author Contributions

All authors listed have made a substantial, direct and intellectual contribution to the work, and approved it for publication.

## Conflict of Interest

The authors declare that the research was conducted in the absence of any commercial or financial relationships that could be construed as a potential conflict of interest.
