# Supplementary material for: International Rickettsia Disease Surveillance: An Example of Cooperative Research to Increase Laboratory Capability and Capacity for Risk Assessment of Rickettsial Outbreaks Worldwide
Source: Front Med (Lausanne). 2021 Mar 2;8:622015. doi: 10.3389/fmed.2021.622015 (PMC7960665; doi:10.3389/fmed.2021.622015)
Supplement: Supplementary file 1 [file Data_Sheet_1.docx]

Supplementary Table 1: Collaborative Rickettsial Investigation Findings by Country, Institutes and Funding Sources

| Host Countries and Institutes | Research Funding Support* | Evidence of Rickettsial Infections** | References |
| --- | --- | --- | --- |
|  |  |  |  |
| Azerbaijan  (Republican Antiplague Station, Baku; Ministry of Defense, Head Medical Office, Baku; Republican Hygiene and Epidemiology Center, Baku, Azerbaijan) | DTRA AJ-TAP-2 | Project title: A seroprevalence study of prior exposure to select arthropod-borne and zoonotic infections among rural populations in three regions of Azerbaijan  Issue: Historical data suggested that rickettsial diseases were endemic to Azerbaijan, but limited current data existed to support the idea.  Methods: A preliminary study was conducted to look at arthropod-borne and zoonotic diseases including rickettsial diseases presence among 796 people from 40 villages in northern Azerbaijan. The serum samples were assessed by TGR and SFGR ELISAs-IgG.  Results: The prevalence of IgG against TGR and SFGR was 0.6% & 15.9%, respectively.  Conclusion: A very low risk among the 40 villages in northern Azerbaijan for typhus group rickettsiosis but a moderate risk for SFGR infections.  Way forward: Additional seroprevalence studies throughout the country should be undertaken, as well as ectoparasites surveillance studies of domestic and peri-domestic animals and arthropod-borne febrile disease hospital-based studies. | 30 |
|  | DTRA AJ-TAP-4 | Project title: A prospective cohort study of the incidence and prevalence of select arthropod-borne and zoonotic infections among Azerbaijani military personnel  Issue: Little serologic evidence of rickettsial infections existed in Azerbaijan.  Methods: Blood was drawn and demographic information collected from enrolled young men twice with a year. TGR and SFGR ELISAs-IgG were used to determine seroprevalence and cumulative seroincidencde of rickettsial infections.  Results: Human serum samples (n=886) were assessed and the seroprevalence was determined to be TGR 0%; SFGR 3.7% (n=33) at enrollment. Twelve months later blood was collected and tested. The seroindicence was determine by assessing seroreactivity of the 853 negative individuals 12 months later. Eighteen individuals seroconverted to give a one year cumulative seroincidience of 2.1%.  Conclusion: Data suggest low risk of rickettsial disease to young men in Azerbaijan.  Way forward: Additional surveillance studies need to be completed in multiple locations within Azerbaijan to confirm low risk or rickettsial infections throughout the country. | 31-33 |
|  | DTRA - RDRP | Project title: Analysis of tick samples from Georgia and Azerbaijan for pathogens.  Objective: Evaluation of 3 newly developed tick-borne *Rickettsia* species-specific qPCR assays utilizing DNA extracted from 15 pools of ticks collected from Azerbaijan rodents.  Methods: Ixodid ticks DNA (n = 15 pools) was assessed by genus-specific qPCR assay (Rick17b), and three species-specific qPCR assays (Raeschl, Rraoul, and Rslov).  Results: 11/15 pooled tick DNA samples were *Rickettsia positive.* All 11 All *Rickettsia* positive samples were negative for *R. aeschlimannii, R. raoultii* and *R. slovaca*. However, 2/11 positive samples were *R. felis* group positive.  Conclusion: Presence of *R. felis* group among ticks in Azerbaijan, but not *R. aeschlimannii, R. raoultii* and *R. slovaca.*  Way forward: Need to assess the 9 *Rickettsia* positive samples with other species-specific qPCR assays and/or conduct MLST to identify the SFGR. Moreover, additional ectoparasite surveys involving not only rodents but domestic and peri-domestic animals need to be done. | 34 |
| Chile  (School of Medicine, Pontificia Universidad  Católica de Chile, Santiago;  Facultad de Ciencias Veterinarias, Universidad Austral de Chile, Valdivia; Clínica Alemana de Santiago Facultad de Medicina Clínica Alemana, Universidad del Desarrollo, Santiago) | FONDECYT,OSU and GEIS | Project title: Evaluation of potential rickettsial disease case in Chile.  Objective: Identify the cause of a potential rickettsial disease case in Chile.  Methods: Clinical blood and eschar samples were collected. Acute and convalescence serum samples were tested for: IgG against STGO, TGR and SFGR. A serum sample (acute) was tested for presence of *htrA* and *groEL* genes by nPCR, qPCR. An eschar sample was assessed by nPCR and sequencing for *htrA* and *groEL* to identify the causative agent.  Results: Acute phase sera negative for IgG STGO, SFGR and TGR. Convalescence serum positive only for IgG against STGO (titer: 400). Eschar sample: *rrs* sequence phylogenetically related to *Orientia* sp.; distantly related to *O. tsutsugamushi*.  Conclusion: 1st case of scrub typhus in South America, 2nd case outside of endemic area of Asia-Australia.  Way forward: Survey for additional cases of scrub typhus and other rickettsial diseases in Chile. Characterize new *Orientia* sp. and South American scrub typhus. Conduct hospital-based study to determine incidence and risk of scrub typhus outbreaks. | 26 |
|  | FONDECYT and GEIS-RDRP | Project title: Assessment of domestic dogs for evidence of rickettsial infection.  Objective: Determine if domestic dogs are susceptible to *Orientia* sp. infection and if so what is the distribution of positive dogs on Chiloé Island, southern Chile.  Methods: Obtained dog serum samples, Chiloé Island (n=202). Perform STGO-ELISA-IgG.  Results: STGO seroprevalence was 21.3%. Positivity more common among older dogs and dogs from rural areas.  Conclusion: As in Asia, dogs in Chile are good sentinels for scrub typhus.  Way forward: Conduct additional serosurveys for both dogs and humans throughout Chile. Also, conduct serosurveys; collect ectoparasites from peri-domestic small animals. | 35 |
|  | FONDECYT and GEIS-RDRP | Project title: Case report of Korean traveler with scrub typhus.  Issue: Traveler from Republic of Korea (RoK) with clinical presentation of scrub typhus needed diagnosis to be confirmed by laboratory studies and to identify, if possible, whether the causative agent was from Korea or Chile.  Methods: Conduct scrub typhus ELISA-IgM (InBios) and IFA-IgG (Fuller Labs) on sera and extract DNA from eschar and perform qPCR and MLST.  Results: Serologies were positive for IgM and IgG against *O. tsutsugamushi* antigens. Eschar sample was positive by Otsu47 qPCR assay for *O. tsutsugamushi*. MLST utilized *rrs, htrA* and *tsa56* and determine the causative agent was *O. tsutsugamushi*, most likely from RoK and not Chile.  Conclusion: Can differentiate scrub typhus cases from Asia-Australia and Chile.  Way forward: Continue to look for scrub typhus cases among international travelers. | 36 |
|  | FONDECYT and GEIS-RDRP | Project title: Distribution of scrub typhus cases in Chile.  Objectives: Confirm identity of scrub typhus clinical cases from continental Chile by lab diagnosis. Determine the distribution of scrub typhus cases in Chile. Ascertain whether scrub typhus cases are limited to only Chiloé Island.  Methods: Evaluate clinical samples (sera and eschars) from suspect scrub typhus cases (n=9) from continental Chile. Use ELISA-IgM & IgG (inBios) and IFA-IgG (Fuller Labs). Assess eschar DNA samples with genus-specific *Orientia* qPCR assay (Orien16S).  Results: ELISA and IFA were positive for all 9 cases with seroconversion among 3 of 6 patients with paired samples. All (9) eschar samples were positive by Orien16S qPCR and by sequencing *rrs* and *htrA* genes.  Conclusion: Scrub typhus is not limited to Chiloé Island but is endemic throughout Chile.  Way forward: Continue to monitor scrub typhus cases in Chile. Follow up with serosurveys of humans, dogs and peri-domestic small mammals. Identify arthropod vectors by evaluating domestic and peri-domestic small mammals and their ectoparasites from locations near cases for evidence of *Orientia* species. | 37 |
|  | FONDECYT and GEIS-RDRP | Project title: Identify potential scrub typhus vectors.  Objective: Determine the vector and vector hosts associated with scrub typhus in Chile.  Methods: Trap peri-domestic small mammals near scrub typhus residences. Detect and identify trapped animals and mite species from small mammals. Assess mites for the presence of *Orientia* with genus-specific qPCR assay (Orien16S).  Results: Rodents (n=244) trapped in 6 rural locations in endemic Chiloé Island were surveyed for mite species. Three species were found (55% prevalence among rodents). One species, *Herpetacarus* sp. was most common (93%) and was the only mite species positive for *Orientia* species (15.8% of pools).  Conclusion: *Herpetacarus* mites are potential reservoirs for *Orientia* sp. causing scrub typhus in Chile.  Way forward: Conduct laboratory vector studies of infected mites. Characterize mites for transstadial and transovarial transmission of the orientiae. Conduct surveys throughout Chile to identify additional small mammal hosts for mites and their mites for orientiae. | 38 |
|  | FONDECYT  and GEIS-RDRP | Project title: Genetically characterize orientiae from scrub typhus cases.  Objective: Determine if scrub typhus case orientiae are diverse genetically like *O. tsutsugamushi* or are the genes sequences conserved.  Methods: Human clinical scrub typhus cases qPCR positive (n=18) were characterized by sequencing *rrs* and *htrA*:  Results: Sequences of *rrs* and *htrA* for all 18 clinical samples were very similar (< 2 nucleotide differences).  Conclusion: Unlike *O. tsutsugamushi*, the *Orientia* species of Chile to date, are not diverse genetically. Moreover, the agents appear to represent a new species of *Orientia*, i.e. *Ca.* O. chiloensis.  Way forward: Continue to assess clinical and environmental orientiae genetically to confirm or refute genetic diversity in Chile. Obtain culture isolates to more completely characterize the proposed new species of *Orientia*. | 24 |
|  | FONDECYT  and GEIS-RDRP | Project title: Conduct serosurvey of rickettsial disease in Chile.  Objective: Conduct a comprehensive serosurvey for rickettsial infections in Chile to determine risk of rickettsial disease.  Methods: A cross-sectional serological survey for antibodies against TGR, SFGR and STGO among healthy adults (n=1302) in rural and urban settings of five regions in northern central and southern Chile was performed.  Results: Seroprevalence of TGR, SFGR and STGO infections were found to be 1.2%, 5.3%, and 0.4%, respectively, among participants.  Conclusion: Rickettsial infections appear to be low in Chile, indicating a low risk of rickettsial disease.  Way forward: Determine the causes of TGR and SFGR infections, by conducting hospital-based fevers of unknown etiology. Survey ectoparasites (ticks, mites, fleas, lice) from domestic and peri-domestic animals for evidence of TGR and SFGR. | 39 |
| Georgia  (National Center for Diseases Control and Prevention, Tbilisi; Laboratory of the Ministry of Agriculture, Tbilisi, Georgia) | DTRA-RDRP | Project title: Analysis of tick samples from Georgia and Azerbaijan for pathogens.  Objective: Evaluate and utilize 3 newly developed qPCR assays for tick-borne rickettsiae with tick samples from Georgia.  Methods: Ticks from Georgia were collected mostly from livestock and some from tick drags (n = 228). DNA from ticks were assessed by a genus-specific (Rick17) and three species-specific (Raeshl, Rraoul and Rslov) qPCR assays.  Results: The minimal infection rates for *R. raoultii and R. slovaca in D. marginatus* were determined to be 10% and 4%, respectively, and for *R. aeschlimannii* in *H. sulcata* and *Hyalomma* spp. to be 1.9% and 20%, respectively, utilizing the new species-specific qPCR assays.  Conclusion: The data indicates the presence and the prevalence of these three rickettsial pathogens among their host ticks in Georgia.  Way forward: Conduct a more comprehensive investigation of tick-borne rickettsiae among ticks collected throughout the country of Georgia. | 34 |
|  | GEIS-RDRP DTRA:GG-21 | Project title: Human disease epidemiology and surveillance of especially dangerous pathogens in Georgia  Objective: Determine the prevalence of rickettsial infections among fever patients in Georgia.  Methods: A hospital-based fever study was conducted to determine etiologies. In this portion of the study we determined the seroprevalence of antibodies against TGR, SFGR, and STGO among enrolled patients (n=655) using acute blood samples and group-specific IgG-ELISAs.  Results: The prevalence of IgG against TGR, SFGR and STGO was 0.3%, 1.5% and 0.3%, respectively.  Conclusion: The seroprevalence of antibodies against rickettsial infection among fever patients was low and thus, the risk among the patient population assessed was low.  Way forward: Conduct serosurveys in other locations in Georgia for evidence of rickettsial infections. In addition, perform arthropod surveys to identify rickettsial agents throughout Georgia. | 40 |
|  | DTRA: GG-TAP-4 and GG-TAP-12 | Project titles: 1) Prevalence of *Rickettsia*, *Ehrlichia,* and *Borrelia* species pathogens in ticks from Georgia; and 2) Analysis of previously identified *Rickettsia* positive Georgia ticks by multi-locus sequence typing.  Objective: Determine the presence, identity, prevalence and distribution of tick-borne rickettsiae across the country of Georgia.  Methods: To more broadly assess the presence of tick-borne rickettsiae from Georgia we examined 1,594 ticks, representing 18 species from five genera (*Ixodes, Hyalomma, Haemaphysalis, Dermacentor*, and *Rhipicephalus*), collected from eight regions of Georgia. A total of 498 tick DNA samples extracted from single or pooled ticks were assessed by molecular methods including Rick17b, Rraoul, Rslov, Raesch, Rmona, Rconor, Rmass9666 qPCR assays and MLST with gltA, ompA, ompB, sca4.  Results: It was found that 9 species of *Rickettsia* (6 human pathogens and 3 species with unknown pathogenicity) were detected from 12 tick species of five different genera throughout the country of Georgia. A distribution map was developed to show the locations of the tick-borne pathogenic rickettsiae.  Conclusion: Six rickettsial pathogens (2 unknown in Georgia) and 3 rickettsiae of unknown pathogenicity (previously unknown in Georgia) were identified, their prevalence/MIR and their distribution discerned.  Way forward: Conduct human serosurveys in the eight regions evaluated to determine more appropriately the risk of the identified rickettsiae in causing infections. | 41 |
| India  (Regional Medical Research Centre, Dibrugarh) | DTRA-RDRP and ICMR | Project title: Assess individuals from Northeast India for seroprevalence of rickettsioses.  Issue: After 67 yrs scrub typhus “returned” to Northeast (NE) India. Unknown was the current risk of scrub typhus as well as other rickettsial diseases in NE India.  Methods: Assess healthy humans (n=1,265) by serologic assays to see how prevalent scrub typhus (STGO-ELISA-IgG), typhus (TGR-ELISA-IgG) and spotted fever (SFGR-ELISA-IgG) in scrub typhus endemic areas of NE India. Extract DNA from serum positive samples and screen by qPCR assays for rickettsiae (Rick17b) and PCR (*tsa56*) orientiae. In addition, assess arthropods (n=131) from domestic and peri-domestic animals to survey for rickettsiae.  Results: Determined that human seroprevalence of 30.8%, 4.2% and 13.8% for STGO, TGR and SFGR, respectively, existed in Northeast India. Two serum samples from STGO positive samples were also positive for *O. tsutsugamushi* by PCR. Rick17b qPCR assay was negative for all ticks and mites assessed, except 4 (out of 16) cat fleas. Sequence of *gltA* showed four *Ca.* R. senegalensis-infected fleas.  Conclusion: These results show that in addition to infection by STGO, individuals were also infected with TGR and SFGR. Presence of a rickettsia in fleas, but not other arthropods suggest a risk to *Ca. R. senegalensis* an incompletely characterized flea-borne agent.  Way forward: Need more complete surveillance of humans, small mammals and arthropod vectors (mites, ticks, fleas, lice) for identification, prevalence-incidence, & distribution of rickettsial infections in Northeast India. | 42 |
|  | DTRA-RDRP and ICMR | Project title: Determine genetic characterization of *Orientia tsutsugamushi* in Northeast (NE) India  Issue: Scrub typhus recently reconfirmed to be endemic for NE India. Unknown is the genetic characterization of the *O. tsutsugamushi* strains currently in NE India.  Methods: Screened fever patients suspected of scrub typhus or FUO with InBios scrub typhus ELISA-IgM. Positive serum samples’ DNA was extracted and PCR amplicons were produced for *tsa56* sequencing*.*  Results: Of 370 patients sera assessed 19.4% were scrub typhus ELISA-IgM positive. Thirteen samples were *tsa56* positive and the amplicons sequence. Phylogenetic analysis showed 3 distinct clades that clustered with stains from India, Taiwan and Thailand.  Conclusion: The first molecular characterization of *O. tsutsugamushi* was determined for NE India.  Way forward: Continue to genotype *O. tsutsugamushi* to ensure that future diagnostics and vaccine candidates include NE India genotypes for enhanced efficacy. | 43 |
|  | DTRA-RDRP and ICMR | Project title: Determine the risk of spotted fever in Northeast India.  Objective: Determine the risk of SFGR in NE India by assessing the seroprevalence of healthy individuals. In addition, ascertain what is/are the SFGR agents.  Methods: In Nagaland, NE India, 462 serum samples from healthy individuals were assessed by SFGR-ELISA-IgG. Positive SFGR-ELISA serum samples were examined by PCR for 17 kDa antigen gene. One sample was positive and was sequenced.  Results: Fifty-one (11%) of 462 serum samples were positive for SFGR-IgG. Only one sample was 17 kDA antigen gene PCR positive. The sequence showed the agent to be *R. felis*.  Conclusion: This was the first evidence of *R. felis* infection in NE India.  Way forward: Need to follow up with additional hospital-based studies in NE India to identify and determine incidence and distribution of SFGR. Moreover, additional arthropod vector surveillance studies need to be performed. | 44 |
|  | DTRA-RDRP and ICMR | Project title: Determine the seroprevalence of typhus group rickettsia in Northeast India.  Issue: Murine typhus is known to exist in NE India, but the prevalence and distribution is not well described. Also, it is unknown the contribution of TGR in acute encephalitis syndrome (AES) and undifferentiated febrile illness in NE India.  Methods: 2,199 patients’ sere were assessed for IgG against TGR-IgG. Those positive were assessed for the presence of the *Rickettsia* genus-specific 17 kDa antigen gene in the patients’ whole blood.  Results: Approximately 2% of samples tested were titer positive TGR-IgG. None of these samples were able to produce a 17 kDa antigen gene fragment for sequencing.  Conclusion: The seroprevalence for TGR in NE India is low but consistently detected. Thus, there is a low risk of murine typhus in NE India.  Way forward: Continue with hospital based studies to characterize murine typhus in NE India. In addition, identify the arthropod vector(s) (*X. cheopis* and other rodent and cat fleas), prevalence and distribution to map out the risk of murine typhus in NE India and compare the data with the rest of India and neighboring countries. | 45 |
| Kazakhstan  (Uralsk Anti-plague Station, Uralsk; Scientific Center of Quarantine and Zoonotic Diseases; and Scientific and Practical Center of Sanitary and Epidemiological Expertise and Monitoring, Almaty) | DTRA: KZ-TAP-2 | Project title: Species identification of tick vectors associated with infectious disease in Kazakhstan  Issue: Ixodid ticks are often hard to identify by morphologic criteria especially the immature life stages. Thus, use of molecular methods would be of benefit to identify efficiently ticks.  Methods: Collect ticks in tick-borne disease endemic Northwest Kazakhstan. Identify ticks by morphologic and molecular characteristics and ascertain the reliability of the molecular assays.  Results: Of 2,232 ticks collected and identified by morphological characteristics 4 genera were correctly identified by molecular assays: *Dermacentor, Hyalomma, Rhipicepahlus* and *Ixodes*.  Conclusion: Use of molecular assays can be used to identify ticks to the genus-level.  Way forward: Develop and evaluate species-specific molecular assays to identify ticks. | 46 |
|  | DTRA: KZ-29 | Project title: The epidemiology of Crimean-Congo hemorrhagic fever, hantavirus (hemorrhagic fever with renal syndrome), and tick-borne viral and rickettsial diseases in the Republic of Kazakhstan  Issue: Tick-borne rickettsia agents such as *R. conorii* subsp. *Cassia* are known to exist in Western Kazakhstan oblast. There does not exist a method to easily screen ticks for rickettsiae in Kazakhstan. Thus, new method to screen for the presence of tick-borne rickettsiae, a genus-specific assay (Rick17b), needed to be evaluated.  Methods: 50 pools of *Rhipicephalus pumilio* ticks (n=180) DNA were extracted. *Rickettsia* presence was determined by the *Rickettsia* genus-specific qPCR assay (Rick17b).  Results: Of 12 tick pools assessed 6 tick pools were positive for *Rickettsia* species.  Conclusion: The Rick17b assay was found to effectively detect tick-borne rickettsiae.  Way forward: Assess the rickettsia-positive tick pools by species-specific qPCR assays and/or MLST to identify the rickettsiae to the species level. Assess other tick species for the presence, distribution, identification and prevalence of rickettsiae in Western Kazakhstan and expand research to include other areas within Kazakhstan. | 47 |
|  |  | Project title: The epidemiology of Crimean-Congo hemorrhagic fever, hantavirus (hemorrhagic fever with renal syndrome), and tick-borne viral and rickettsial diseases in the Republic of Kazakhstan  Issue: Tick-borne rickettsial diseases are known to exist sporadically in Kazakhstan. However, they were unknown to exist in Karaganda oblast.  Methods: Identify presence of rickettsiae within *Dermacentor marginatus* collected by tick drags using genus-specific qPCR assay (Rick17b).  Results: Of 33 tick pools (n=330) assessed 2 tick pools were positive for *Rickettsia* species (0.6% MIR).  Conclusion: In the Karaganda oblast it was determined for the first time the presence of tick-borne rickettsiae.  Way forward: Assess the rickettsia-positive tick pools by species-specific qPCR assays and/or MLST to identify the rickettsiae to the species level. Assess other tick species for the presence, distribution, identification and prevalence of rickettsiae in Karaganda and expand research to include other areas within Kazakhstan. | 48 |
|  |  | Project title: The epidemiology of Crimean-Congo hemorrhagic fever, hantavirus (hemorrhagic fever with renal syndrome), and tick-borne viral and rickettsial diseases in the Republic of Kazakhstan  Objective: Complete a more comprehensive tick-borne survey to confirm and expand previous limited tick-borne rickettsial investigations.  Methods: Utilize 2,358 ticks collected from animals and tick drags by three anti-plague stations in southern Kazakhstan for presence of rickettsiae. DNA extracts from 150 tick pools were assessed initially by *Rickettsia* genus-specific qPCR assay (Rick17b). Those samples positive were further assessed with three *Rickettsia* species-specific assays (Raesch, Rraoul, and Rslov).  Results: Of 150 pools 27 were positive for *Rickettsia*. Of these 6, 5, 2 and 14 were identified as *R. slovaca*, *R. raoultii, R. aeschlimannii* and *Rickettsia* spp., respectively.  Conclusion: Three pathogenic species of *Rickettsia* were identified in southern Kazakhstan. In addition, 14 of 27 pools of ticks were not identified to species level.  Way forward: Additional species-specific assays and/or MLST are needed to identify other tick-borne rickettsiae in southern Kazakhstan. Similar studies need to be completed across Kazakhstan as well as studies of healthy and febrile individuals to ascertain the risk of tick-borne rickettsial diseases in Kazakhstan. | 49 |
|  | DTRA: CAP-1 & KZ-31 | Project titles: 1) Flea-borne disease surveillance; and 2) Effect of *Rickettsia* spp. upon fitness of *Yersinia pestis* in fleas that vector plague in the Republic of Kazakhstan.  Objective: Ascertain whether flea-borne rickettsiae are found among fleas known to vector *Yersinia pestis*, the causative agent of plague.  Methods: DNA from 2,963 fleas associated with Giant Gerbils in Almaty oblast were assessed by genus- and species-specific qPCR assays for flea-borne rickettsiae.  Results: Flea-borne rickettsiae were identified as *R. asembonensis, R.felis/Ca*. R. senegaleonsisi, and *R. typhi* at prevalences of 41.1%, 10.5% and 0%, respectively.  Conclusion: Flea-borne rickettsiae were detected among 69.5% of fleas known to vector plague that were collected from rodent hosts of plague.  Way forward: To conduct a study to determine the impact, if any, on the presence of flea-borne rickettsiae with the fitness of *Y. pestis* within the flea vector of plague. | 50 |
| Madagascar (Institut Pasteur de Madagascar, Antananarivo) | GEIS-RDRP and IPM | Project title: Flea-borne rickettsial diseases in Madagascar.  Issue: Flea-borne plague is endemic for Madagascar. Unknown is the presence of other flea-borne diseases such as those caused by rickettsiae in Madagascar.  Methods: To ascertain whether flea-borne rickettsial diseases were endemic to Madagascar a two-site (urban and rural) randomized study was conducted. From each site 31 adults were enrolled and assessed for TGR and SFGR IgG by ELISAs. Peri-domestic small mammals were collected in house and around houses from each site. Blood and fleas were collected from trapped animals. Serum was assessed by TGR- and SFGR-ELISAs-IgG. Additionally, fleas were trapped in study homes by light traps. Fleas were assessed for molecular evidence or rickettsial DNA.  Results: For humans 39% and 34% of their blood samples were positive for IgG against TGR and SFGR, respectively. The prevalence for antibodies to each of the two groups of rickettsiae between the two sites was not significantly different. Thus, both groups had similar prevalence of infection with rickettsial agents from the same groups. Among 46 small mammals captured only two were positive for IgG against TGR (and none positive for IgG against SFGR). Fleas (n=143) from small mammals and light traps positive for *R. typhi* (18.2%) and *R. felis* (7.0%).  Conclusion: These results show that two *Rickettsia* species pathogens, *R. typhi* from TGR and *R. felis* from SFGR were found among peri-domestic small mammals and with in homes. The high prevalence of antibodies to these two groups of rickettsiae in humans in the same sites suggest they may be due to flea-borne rickettsiae.  Way forward: Conduct hospital-based studies to determine the identity of agents causing the high prevalence of rickettsial infections found in this study, and determine incidence and distribution of these diseases. | 51 |
|  |  |  |  |
|  |  |  |  |
| Thailand  (Khon Kaen University, Khon Kaen) | GEIS-RDRP and KKU | Project title: Role of companion cats and cat fleas play in rickettsial diseases in Northeast Thailand.  Objective: To determine the role that companion cats and cat fleas play in rickettsial diseases in Northeast Thailand.  Methods: Assess cats for serological evidence of rickettsial infections using SFGR-ELISA-IgG. Assess presence of cat flea-borne rickettsiae with genus-, group- and species-specific qPCR assays, and confirm identity by sequencing *ompB* fragment.  Results: Two of 42 cat sera assessed were seropositive for SFGR. Of 23 cat fleas from 3 dogs 21 were positive for *R. asembonensis*-an agent suspected to be pathogenic for humans, non-human primates and dogs.  Conclusion: Infection of companion cats and cat fleas from companion animals with a SFGR suggest a risk to humans of infection with one or more SFGR in NE Thailand.  Way forward: Conduct more complete surveillance of companion animals and their ectoparasites for the presence of rickettsial infections throughout Northeast Thailand. Assess presence of IgG against SFGR, TGR and STGR among healthy (single serum) and febrile patients (with paired serum samples). | 52 |
|  |  |  |  |
|  |  |  |  |
| Ukraine  (Lviv Scientific Research Institute of Epidemiology and Hygiene, Lviv) | DTRA PDG for UP-1 | Project title: Evaluation of arthropod-borne infections in Ukraine.  Issue: In western Ukraine in Lviv oblast epidemic typhus was once endemic (1922-37), so to assess the current risk of TGR diseases the seroprevalence of antibodies against TGR was performed.  Methods: 1,000 healthy people enrolled from 2 districts were assessed for IgG against TGR by ELISA.  Results: Seventeen (1.7%) individuals had IgG against TGR  Conclusion: The seroprevalence of antibodies against TGR was only 1.7%, suggesting that the risk of epidemic typhus outbreaks occurring from folks previously infected with *R. prowazekii* (Brill-Zinsser disease) is very low. Moreover, it is unknown with the group-specific antibodies detected whether the antibodies were produce by infection with *R. prowazekii* or *R. typhi-*the causative agent of murine typhus.  Way forward: To confirm the low risk of epidemic typhus, a survey to look for human body lice among the population and assessed whether they are infected with *R. prowazekii* needs to be performed. In addition, a survey of rodent-borne fleas for the presence/absence of *R. typhi* should be conducted. | 53 |
|  |  | Project title: Evaluation of arthropod-borne infections in Ukraine.  Objective: To determine the seroprevalence in western Ukraine of SFGR infections was unknown though they are common around the world.  Methods: Utilize the same two populations from above (1,000 healthy people enrolled from 2 districts in Lviv Oblast) to determine the seroprevalence for IgG against SFGR by ELISA.  Results: 5.1% of individuals were seropositive for SFGR.  Conclusion: This suggests a moderate risk of SFGR diseases among the population. The SFGR responsible for these infections are unknown.  Way forward: Survey domestic and peri-domestic animals for ectoparasite-borne SFGR (i.e. ticks, mites and fleas) to identify pathogenic SFGR in the two locations surveyed. | 54 |
| Vietnam  (Hanoi Medical University and National Hospital for Tropical Diseases, Hanoi) | NAFOSTED | Project title: The presence and prevalence of rickettsial infections among humans in northern Vietnam.  Objective: To determine the presence and prevalence of rickettsial infections among humans in northern Vietnam.  Methods: Utilize group-specific ELISAs-IgG to detect evidence of TGR, SFGR and STGO infections among 908 residents from rural and urban northern Vietnam.  Results: Seroprevalences of TGR, SFGR and STGO were 6.5%, 1.1% and 1.7%, respectively. More infections of TGR in occurred in urban areas than rural areas.  Conclusion: Low prevalence and therefore low risk of rickettsial infections among people residing in northern Vietnam.  Way forward: Determine cause of rickettsial infections by assessing domestic and peridomestic animals and their ectoparasites. In additions, assess presence, identity, distribution and incidence of rickettsial diseases in northern Vietnam and subsequently all of the country. | 55 |
|  | DTRA-RDRP and NAFOSTED | Project title: Characterize clinical manifestations of rickettsial diseases and determine the applicability of molecular assays in rickettsial diagnosis.  Objectives: To characterize clinical manifestations of rickettsial diseases not clearly defined by previous studies. In addition, ascertain the applicability of molecular assays in rickettsial diagnosis.  Methods: Enrolled 302 febrile patients suspected of rickettsioses. Use patients’ case report forms to collect clinical & laboratory information. Utilize and determine benefit of serological and molecular assays to confirm clinical diagnoses.  Results: Confirmed 34.1% and 3.3% of patients by clinical presentation, serologies and molecular assays for scrub typhus and murine typhus, respectively.  Conclusion: These results highlight the need for increased emphasis on training for healthcare providers for earlier recognition (especially in identifying eschars), prevention, and treatment of rickettsial diseases in Vietnam.  Way forward: Increase training of health care providers in clinical assessment of febrile patients and laboratorians in use of serological and molecular assays to diagnose rickettsial diseases in Vietnam. In addition, conduct a country-wide study to determine the presence, distribution, and incidence of rickettsioses and determine their risk in Vietnam. | 56 |
|  |  |  |  |
|  | DTRA-RDRP and NAFOSTED | Project title: Determine the genetic makeup of *O. tsutsugamushi* causing scrub typhus in Vietnam.  Objective: To ascertain the genetic makeup of *O. tsutsugamushi* causing scrub typhus in Vietnam. Knowing the genotypes present is necessary information for the development of diagnostics and vaccines appropriate for Vietnam.  Methods: Utilize sequencing and phylogenetic analysis of the variable 56-kDa type-specific antigen gene (*tsa56*) fragment to genotype *O. tsutsugamushi* in Vietnam.  Results: Of 116 positive samples, 65 provided *tsa56* sequences that by phylogenetic analysis were classified into 3 geno-groups: Karp, Kato and Gilliam.  Conclusion: Scrub typhus diagnostics and vaccines should be developed utilizing these results for Vietnam.  Way forward: Conduct similar studies throughout Vietnam to determine the prevalence and distribution of the most common genotypes. Develop and assess new molecular diagnostics and vaccine candidates for scrub typhus use in Vietnam. | 57 |
|  |  |  |  |
|  |  |  |  |
|  |  |  |  |
